# Women’s knowledge and associated factors on preconception care at Public Health Institution in Hawassa City, South Ethiopia

**DOI:** 10.1186/s13104-018-3951-z

**Published:** 2018-11-28

**Authors:** Andargachew Kassa, Zemenu Yohannes

**Affiliations:** Department of Midwifery, College of Medicine and Health Sciences, Hawassa University, Hawassa, Ethiopia

**Keywords:** Knowledge, Preconception care, Ethiopia

## Abstract

**Objective:**

Preconception care is pivotal to improve pregnancy and birth outcome. It is vital for the future health of mother, her child and her family, which is routinely practice. The study aims to assess knowledge of preconception care and associated factors in post natal women at public health institution in Hawassa city, South Ethiopia.

**Results:**

In this study 20% (95% CI 16.9, 23.1) of post natal women at public health institution had a good level of knowledge on preconception care. Women who have secondary and above education level, urban residence, and have at least one ANC contact had significantly higher odds of good level of knowledge on preconception care. The finding of this study showed that level of women’s knowledge towards preconception care to be low compared to other studies. Having at least one ANC contact, urban residence and having secondary and above education are predictors of knowledge on preconception care. It shall be beneficial if the city health administration, regional and national health authorities work towards improving the knowledge of mothers towards preconception care as well as routine provision of preconception care in the health care system.

## Introduction

Preconception care (PCC) is pivotal to improve pregnancy and birth outcome. It is vital for the future health of mother, her child and her family, preconception care (PCC) is used. Despite policy planners and stakeholders are given priority agenda for maternal and child health care, maternal and neonatal mortality reduction is not at the desired level [1, 2]. Preconception health care hasn’t become part of routine practice across the globe. In low income countries, the implementation of PCC is almost nil. Nevertheless in this region, there is higher maternal and neonatal death of which 90% are preventable [3–6].

Maternal and child health experts recommend that preconception health care is an essential intervention to modify biomedical, behavioural and social risks for better pregnancy and child birth outcome through risk assessment, health promotion, disease prevention and care provisions [7, 8]. Preconception care (PCC) is the provision of biomedical, behavioural and social health interventions to women and couples before conception and aims at improving their health status, and reducing behaviours and individual and environmental factors that contribute to deprive maternal and child health outcomes [9].

In 2015, an estimated 303,000 women died as a result of pregnancy and child birth related complications in the world, of which 99% occurred in low and middle-income countries, especially south Asia and sub-Saharan Africa. The frontline cause of maternal deaths globally are hemorrhage (27%), pre-existing medical conditions (15%), hypertension (14%), sepsis (11%), abortion (8%), and other indirect causes (7%) [10]. Under-five mortality in 2015 was 42.5 per 1000 live births [11, 12]. In Ethiopia maternal mortality ratio is estimated 412 deaths per 100,000 live births in 2016 and the under-five mortality at 67 per 1000 live birth in 2016 [13].

Preconception care is cardinal to alleviate different risk behaviours, exposures that affects conception, fetal development, and ultimately reduce subsequent adverse outcomes [14]. It is also important for different behavioural changes like maintaining normal body mass index, taking appropriate diet (daily vegetables and fruits), avoiding drinking alcohol, stop smoking, being away from hazardous area, and also attending to early a medical checkup to optimize maternal and neonatal health. For example, risk of neural tube defect can be lessened through supplementation of folic acid 3 months before conception. During the first 7 weeks of gestation (before 52 days of pregnancy) exposure to alcohol, tobacco and other drugs, lack of essential vitamins (e.g. folic acid) and workplace hazards can adversely affects pregnancy outcome and maternal and neonatal wellbeing [15, 16].

Knowledge of preconception care can be obtained from experience, health care providers, coffee ceremony (neighbour meeting to drink coffee and discuss any agenda during that time), family, reading books, newspapers, radio, television, and social media. Studies revealed that women who received pre-pregnancy care have more knowledge and often show risk alleviation behaviours [17].

Studies recommended that antenatal care should start before conception to alleviate bad obstetrics outcome. Maternal and child health planners, policy-makers and stakeholders should be cognizant of the values of PCC to attain the sustainable development goal (SDG) targets in relation to maternal, neonatal and child health. Therefore, evidence of knowledge and associated factors towards preconception care among mothers in Ethiopia is very rare. Hence, the aim of this study was to assess the level of knowledge and associated factors towards preconception care among mothers who gave birth at public health institution in Hawassa city, Southern Ethiopia.

## Main text

### Methods and materials

#### Study design and setting

Health institution based cross sectional study was carried out from March 01–30, 2017 among mothers who gave birth at public health institution in Hawassa city. Hawassa is administrative city of Southern Nations, Nationalities and Peoples Regional State (SNNPRS) and located 275 km South from Addis Ababa. According to the 2017 City Health Department estimation report, there were 359,358 people living in Hawassa. The city has 8 sub city and 32 kebeles, which have 83 public and private health institutions. These are one public referral and teaching hospital, one public general hospital, 4 private primary hospitals, 9 public health centers, 17 health posts and 51 private clinics. During the study period, there were 1452 health professionals working in the randomly selected public health institutions of the city.

Hawassa University referral and teaching hospital is the largest hospital in southern Ethiopia with more than 300 beds which renders service in the region and the neighbouring region. The outpatient department consists of 17 rooms and inpatient service which consisted of 5 main departments. The average number of patient flow at the OPD was more than 200 people per day. Hawassa University referral and teaching hospital and Adare general hospital are public health hospitals providing comprehensive essential obstetric care in the City. The remaining 9 PHC are giving basic essential obstetric care. In the past 6 months of the year 2016, there were a total of 4780 deliveries reported from Hawassa University referral and teaching hospital (2073), Adare general hospital (2022), Adare health center (313), Millennium health center (311), and Tilte health center (61).

The source populations were all pregnant women who live in the Hawassa City Administration. Whereas, the study populations were those who gave birth during the study period at public health institution in Hawassa city.

Sample size was determined using single population proportion formula with confidence interval 95%, p-valve 0.5, margin of error (α = 0.05), a design effect of 2 and 10% non-response rate. The total sample size is 580.

Five public health institutions were selected using simple random sampling technique. Multistage sampling technique was used to select a total of 580 study participants using random sampling methods. The calculated sampling interval (K) was 1.2. Based on the finding we consecutively recruited the study subject’s. The samples were taken proportionate to the number of expected deliveries from each selected PHIs. All participants included in the study were all consented to participate wilfully in the study.

The questionnaire was developed by reviewing different existing literatures. First developed in English and translated into Amharic and then back to English to check the accuracy. The socio-demographic factors are; age, parity, educational status, religion, occupation, partner’s occupation, and residence, gravidity, antenatal care attendee, gestation, previous history of adverse pregnancy outcomes such as history of baby with macrosomia and also history of pregnancy induced hypertension in the previous pregnancy, whereas dependent variable was the ‘Women’s Knowledge about PCH/C”.

Before the actual data collection, the questionnaire was tested on 10% care in post natal women at public health institutions in Shashemene city, which is 20 km away from Hawassa city.

Operational definitions: Women’s knowledge about preconception health and care was measured based on the individual study participant’s correct response of 17 items measuring their knowledge about PCC. Each question had one correct answer those who scored 50% and above of the items are labelled as women with “good PCH/C knowledge” whereas those remaining categorized as women with poor PCC knowledge.

Data were collected by 5 BSC female midwives and 5 BSC female nurses after 1-day training about informed consent, techniques of interviewing, and data collection procedures. Two health officers were assigned to supervisors for the data collectors.

#### Statistical analysis

The data were entered and cleaned using SPSS version 20 for analysis. Those factors found with their *P* value ≤ 0.20 in the bi-variable logistic regression model were fitted into the multivariable logistic regression model to control the effect of confounding variables. Multivariable analysis was carried out to evaluate the independent effect of each covariate on ‘good PCH/C knowledge’ by controlling the effect of others. P value < 0.05 is taken as statistically significant. For further analysis, descriptive statistics like frequencies and cross tabulation were performed. Tables and figure were used to present the findings of the study.

### Result

#### Socio demographic characteristics

This study included 580 women who gave birth in public health institutions of Hawassa city. Most of the study participants were ethnic Sidama (40%), followed by Oromo (19.8%), and Wolayita (18.3%). One-fifth (20%) of the women never attended formal education and more than half (56%) attended primary level of education. About two-third (64.7%) of participants were urban residents (Table 1).

**Table 1:** Socio-demographic characteristics of women who gave birth in public health institutions of Hawassa (n = 580), Northern Ethiopia 2017

S. N.	Variables	Categories	Frequency(n)	Percent (%)
1	Age of the mother	< 20 years	45	8
		20–34 years	492	84.6
		35–49 years	43	7.4
2	Marital status	Married	560	96.6
		Single	20	3.4
3	Ethnicity	Sidama	232	40
		Amhara	42	7.2
		Gurage	46	7.9
		Oromo	115	19.8
		Wolayta	106	18.3
		Silte	39	6.8
4	Women’s education status	No formal education	110	19
		Primary education (1–8 grade)	325	56
		Secondary education (9–12 grade)	97	16.7
		Tertiary (college or university)	48	8.3
5	Occupation	Housewife	399	68.8
		Private business	87	15
		Daily worker	14	2.4
		Salaried employed	61	10.5
		Student	19	3.3
6	Monthly income	< 1000 ETB	174	30
		1001–2000 ETB	119	20
		2001–3675 ETB	142	25
		> 3675 ETB	145	25
7	Total family size	< 5	456	78.6
		6-May	89	15.4
		> 6	35	6
8	Residence	Urban	375	64.7
		Rural	205	35.3

#### Prevalence of PCC knowledge

**Fig. 1:**
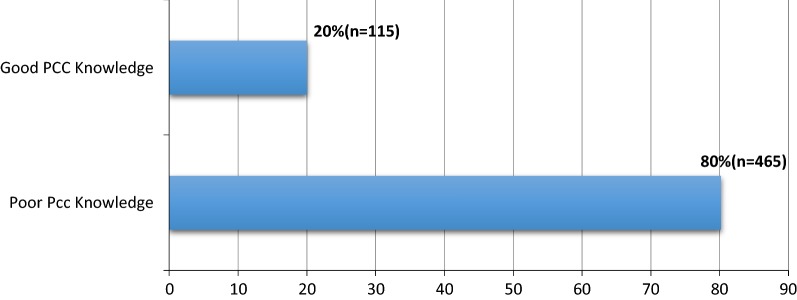
Women’s knowledge about preconception health/care in Hawassa, South Ethiopia, 2017

In this study 20%, 95% CI (16.9, 23.1) has good knowledge about preconception care among women who gave birth in the public health institutions of Hawassa city administration (Fig. 1).

#### Information about preconception health and care

Nearly all of the study participants reported that they never received information regarding preconception care. About one-third 182 (31.4%) of the study participants reported that they get advise on diet/nutrition prior to pregnancy, but only 9 (1.6%) of the study participants reported they were counselled about preconception folic acid supplementation. The other reported preconception care was advice about avoidance of alcohol. This was a case reported by only one participant (0.2%).

The analysis of this study also denoted, next to health professionals, mass media (Television and radio) (4%), Flier or brochures (1.6%), family or friend (2.2%), school or university (1.0%), and internet (0.2%) as source of information preconception health care.

#### Factors associated with women’s knowledge about preconception care

Results of the multivariable logistic regression showed that women who attended to at least one antenatal care clinic contact were four times knowledgeable about preconception care than counterpart (AOR = 4.0, 95% CI 1.1–12.6). Women who live in urban were two times knowledgeable about preconception care than counterpart (AOR = 2.0, 95% CI 1.1–3.3). Those women who attended high school and above were by two times knowledgeable about PCC than their counterparts (AOR = 2.0, 95% CI 1.1–3.3) (Table 2).

**Table 2:** Bivariate and multivariate analysis depicting factors associated with good preconception health/care knowledge among women giving birth in Hawassa, Ethiopia 2017

		Client’s preconception knowledge		COR, 95% CI	AOR, 95% CI
		Poor PCC knowledge	Good PCC knowledge		
Women’s educational status	Elementary and below	372 (64%)	63 (11%)	1	1
	High school and above	93 (16%)	52 (9%)	3.3 (2.1–5.1)***	2.0 (1.1–3.3)*
Husband’s educational Status	Elementary and below	295 (51%)	47 (8.1%)	1	1
	High school and above	170 (29.3%)	68 (11.7%)	2.5 (1.7–3.8)***	1.3 (0.7–2.3)
Monthly house hold income	≤ 1000.0 birr	147 (25.4%)	27 (5.0%)	1	1
	1001.0–2000.0 birr	98 (16.9%)	21 (3.6%)	1.2 (0.6–2.2)	1.4 (0.8–2.9)
	2001.0–3675.0 birr	117 (20.2%)	25 (4.3%)	1.2 (0.6–2.1)	1.1 (0.6–2.1)
	≥ 3676.0 birr	103 (17.8%)	42 (7.2%)	2.2 (1.3–3.9)*	1.5 (0.8–2.9)
Women’s place of residence	Rural	183 (31.6%)	22 (3.8%)	1	1
	Urban	282 (48.6%)	93 (16.0%)	2.7 (1.7–4.5)***	2.0 (1.1–3.3)*
Attended ANC for at least one visit	Not attended ANC	56 (9.7%)	3 (0.5%)	1	1
	Attended at least one ANC visit	409 (70.5%)	112 (19.3%)	5.1 (1.6–16.6)*	4.0 (1.1–12.6)*
${\text{Hx}}^{\smallint }$of previous infant with macrosomia	No history	414 (71.4%)	109 (18.8%)	1	1
	Yes	51 (8.8%)	6 (1.0%	0.4 (0.9–1.1)	0.4 (0.2–1.0)
Hx of pregnancy Induced Hypertension	No	452 (77.9%)	107 (18.5%)	1	1
	Yes	13 (2.2%)	8 (1.4%)	2.6 (1.1–6.4)*	1.7 (0.6–4.4)

$${\text{Hx}}^{\smallint }$$: history

*p < 0.05, **p < 0.01 and ***p < 0.001

### Discussion

Preconception care is a key means for reducing and preventing maternal and child morbidity and mortality. Nevertheless, it is not well practice in developing countries. This study showed that overall level of knowledge on preconception care among mothers who gave birth at public health institution in Hawassa city is 20%. This finding is higher than the study done in Nigeria (2.5%) [18], Iran (10.4%) [19] and Sudan (11.1%) [20]. The higher level of knowledge noticed in this study might be due to the fact that the current study took place immediately after the mothers gave birth.

On the other hand, it is lower than compared to other study done in Amhara region, northern Ethiopia (27.5%) [21], Saudi Arabia (37.9%) [22], United Arab Emirates (46.4%) [23], and Turkey (46.3%) [24], in Kelantan, Malaysia (51.9% [25], Qatar (53.7%) [26], Canada 70% [27], British Colombia 71% [28], in the USA among low income Mexican–American group (76%) [29], Saudi Arabia (84.6%) [30], Jordan (85%) [31].

The low knowledge identified level in this study might be due to the low socioeconomic conditions, due to low media coverage regarding preconception care, low habit of check-up for ANC, the low attention given to preconception care implementation by the health industry across the country, and lack of preconception clinic at health institution level.

In this study there was association of level of knowledge on preconception care with factors such as, level of education, place of residence, and ANC contact. Women who had above secondary education level had two times higher odds to have good level of knowledge on preconception care. This finding is consistent with the study done Ethiopia, Nigeria, Iran, Sudan, USA, Netherlands and Sri Lanka [18, 20–22, 29, 32, 33]. This may be explained as the women’s educational status is increase, their health seeking behabour regarding preconception care will also increase. The more educated women might be eager to know about her own health status, and risks factors leading to ill health. These group of women do have better complication readiness plans. The more educated women might have interest to ask, read, listen, and watch any information sources related to her wellbeing.

Women who live in urban were two times higher odds to have good level of knowledge on preconception care than rural residents. This inconsistency is probably due to the fact that residents living in urban area might have better access to media and health institution. Women who attended at least one ANC contact had by four times higher odds to have good level of knowledge on preconception care. During ANC contact, women are informed about their health status, ways of disease prevention and health promotion, and birth preparedness and complication readiness.

### Conclusion

The finding of this study showed that level of women’s knowledge towards preconception care to be low compared to other studies. Having at least one ANC contact, urban residence and having secondary and above education are predictors of good level of knowledge on preconception care. It would be beneficial if the city health administration, regional and national health authorities work towards improving the knowledge of mothers towards preconception care as well as routine provision of preconception care in the health care system.

## Limitation

One possible limitation of this study is the fact that it didn’t include the husbands or spouses of the women. Results could also be to some extent affected by social desirability biases.

## Abbreviations

ANC: antenatal care
AOR: adjusted odds ratio
BEmONC: basic emergency obstetrics and new-born care
